# 1705. Comparative Analysis of Luminometry -vs- Fluorescent Marker Use for Cleaning and Disinfection Processes in a Pediatric Hospital

**DOI:** 10.1093/ofid/ofad500.1538

**Published:** 2023-11-27

**Authors:** Juan Pablo Londono-Ruiz, Diego Andres Cruz-Acevedo, Lina Paola Gaitan-ayala, Liliana Prieto-Villarraga, Claudia Patricia Correa-Castro, Ivan Felipe Gutierrez-Tobar

**Affiliations:** Clinica Infantil Colsubsidio, Staphylored Colombia, Bogota, Distrito Capital de Bogota, Colombia; Universidad del Rosario, Bogota, Distrito Capital de Bogota, Colombia; Clinica Infantil Colsubsidio, Bogota, Distrito Capital de Bogota, Colombia; Clinica Infantil Colsubsidio, Bogota, Distrito Capital de Bogota, Colombia; Clinica Infantil Colsubsidio, Bogota, Distrito Capital de Bogota, Colombia; Clínica Infantil Santa Maria del Lago y Clinica Infantil Colsubsidio, Bogota, Distrito Capital de Bogota, Colombia

## Abstract

**Background:**

The cleaning and disinfection process (CDP) of hospital surfaces has proven to be an effective tool in preventing healthcare-associated infections. ATP bioluminescence meters have been established as the standard for CDP evaluation. The use of fluorescent markers has also been established as an alternative to this. The aim of this study was to assess the concordance between luminometry measurements and fluorescent marker use in the CDP of a pediatric hospital.

**Methods:**

A diagnostic test study was conducted in which luminometry (Clean-Trace™ LX25 - 3M™) was compared with the use of a fluorescent marker (West Scan - West Química). Joint relative light units (RLU) were taken, and a subjective scale was used to define the erasure of the fluorescent marker after the cleaning and disinfection process (completely erased, partially erased, and intact). The process was established as adequate when less than 65 ATP units were obtained in critical sites and less than 175 in general hospitalization sites. The R program v 4.2.2 was used for data analysis.

**Results:**

We performed 292 paired measurements: 222 (76%) in critical areas and 70 (24%) in non-critical areas. Measurements were taken from different high-contact surfaces such as crash carts, monitor screens, worktables, dressing carts, and defibrillators, among others. The luminometries carried out in 244 (83.5%) were adequate, and the measurements with fluorescent marker showed that 187 (64%) presented total erasure, 37 (12.6%) showed partial erasure, and 66 (22%) were intact. The Kappa index between both tests for total erasure was calculated as adequate, being 0.155 (p-value 0.002). When the tests with the criteria of partial or total erasure were compared as adequate, the Kappa index was 0.007 (p-value 0.6). When they were sub-analyzed according to the areas (critical and non-critical), a kappa value of 0.141 (p-value 0.02) was found for critical areas and 0.194 (p-value 0.03) for non-critical areas.

**Conclusion:**

The correlation between the evaluation of the cleaning and disinfection process using a luminometer and a fluorescent marker showed slight agreement for total erasure. More studies are required to understand the reasons for this low correlation.

**Disclosures:**

**All Authors**: No reported disclosures

